# Palm Kernel Cake Oligosaccharides Acute Toxicity and Effects on Nitric Oxide Levels Using a Zebrafish Larvae Model

**DOI:** 10.3389/fphys.2020.555122

**Published:** 2020-09-24

**Authors:** Rui Qing Foo, Syahida Ahmad, Kok Song Lai, Zulkifli Idrus, Khatijah Yusoff, Juan Boo Liang

**Affiliations:** ^1^Institute of Tropical Agriculture and Food Security, Universiti Putra Malaysia, Kuala Lumpur, Malaysia; ^2^Faculty of Biotechnology and Biomolecular Sciences, Universiti Putra Malaysia, Kuala Lumpur, Malaysia; ^3^Health Sciences Division, Abu Dhabi Women’s College, Higher Colleges of Technology, Abu Dhabi, United Arab Emirates; ^4^Office of the Deputy Vice Chancellor (Research & Innovation), Universiti Putra Malaysia, Kuala Lumpur, Malaysia

**Keywords:** optical transparency, *in vivo*, diaminoflourescein, lipopolysaccharide, mannanoligosaccharide, fructooligosaccharide

## Abstract

One of the beneficial effects of non-digestible oligosaccharides (NDOs) is their anti-inflammatory effects on host animals. While conventional animal studies require that analysis be done after samples have been taken from the host, zebrafish larvae are optically transparent upon hatching and this provides an opportunity for observations to be made within the living zebrafish larvae. This study aimed to take advantage of the optical transparency of zebrafish larvae to study the nitric oxide (NO) reducing effects of NDOs through the use of lipopolysaccharide (LPS) from *Salmonella enterica* serovar (ser.) Enteritidis (*S*. Enteritidis) to induce cardiac NO production. Prior to running the above experiment, an acute toxicity assay was conducted in order to determine the appropriate concentration of oligosaccharides to be used. The oligosaccharides tested consisted of oligosaccharides which were extracted from palm kernel cake with a degree of polymerization (DP) equal to or less than six (OligoPKC), commercial mannanoligosaccharide (MOS) and commercial fructooligosaccharide (FOS). Acute toxicity test results revealed that the OligoPKC has a LC_50_ of 488.1 μg/ml while both MOS and FOS were non-toxic up to 1,000 μg/ml. Results of the *in vivo* NO measurements revealed that all three NDOs were capable of significantly reducing NO levels in LPS stimulated zebrafish embryos. In summary, at 250 μg/ml, OligoPKC was comparable to MOS and better than FOS at lowering NO in LPS induced zebrafish larvae. However, at higher doses, OligoPKC appears toxic to zebrafish larvae. This implies that the therapeutic potential of OligoPKC is limited by its toxicity.

## Introduction

Non-digestible oligosaccharides (NDOs) are low molecular weight carbohydrates that are not enzymatically hydrolyzed and absorbed in the upper gastrointestinal tract of most animals; excluding ruminants which are foregut fermenters (Flint et al., 2012). Rather, they are broken down and utilized by the anaerobic microbes in the colon (Delzenne and Roberfroid, 1994). However, some NDOs are only able to be fermented by a specific group of microbes in the colon and this gives them a competitive advantage over other microbes in the population. This selectivity will inherently lead to a change in the microbial population as well as activity amongst the gastrointestinal microbes and if this change is to the benefit of the host, then the oligosaccharides are termed as prebiotics (Gibson et al., 2010).

A change in gut microbial population will subsequently be detected and reciprocated through the antigen sampling mechanism of the gut-associated lymphoid tissue (GALT) (Murphy, 2011). Using the prebiotic fructooligosaccharide (FOS) as an example, FOS is proven to be selectively fermented by beneficial microbes of the gut, namely microbes of the Lactobacillus and Bifidobacterium species (Kaplan and Hutkins, 2000). The increase in probiotics such as Lactobacillus and Bifidobacterium has been linked to the increase in IgA which modulates gut microbiota and has been reported to possess anti-inflammatory effects (Peterson et al., 2007; Kandasamy et al., 2014).

The control of inflammation in the gastrointestinal tract is of utmost importance. This is because chronic inflammation of the gut may lead to a compromised epithelial barrier which in turn allows for pathogenic and opportunistic microbes to translocate through and this further exacerbates the inflammation (Beeching et al., 2011; Brandl and Schnabl, 2015). One of the markers of inflammation is nitric oxide (NO) which; when present in elevated levels over an extended period of time, can be detrimental to one’s health (Kolios et al., 2004; Avdagić et al., 2013).

Studies have shown that the inclusion of prebiotics were capable of reducing inflammation through the increase in production of IgA and anti-inflammatory cytokines such as IL-4 and IL-10 which has been reported to inhibit NO production (Gazzinelli et al., 1992; Hosono et al., 2003; Kolios et al., 2004; Ogando et al., 2004; das Graças Vaz-Tostes et al., 2014). Conventional methods of measuring NO requires sampling from body fluids such as serum and urine or through gasses produced such as exhaled NO or rectal NO (Goggins et al., 2001; Ljung et al., 2006; Ghasemi et al., 2008; Protopapas et al., 2016). However these methods require that the samples be taken and measured outside the living organism in question.

With zebrafish (*Danio rerio*) as a model organism however, it is possible to observe NO levels in response to inflammation or stress *in vivo*. This is because the zebrafish larvae up to 5 days old are optically transparent and this transparency can be prolonged by treating the embryos with 1-phenyl2-thiourea (PTU) (Karlsson et al., 2001; Chávez et al., 2016). Using a diaminoflourescein probe and a fluorescence microscope, the NO levels measured within the zebrafish larvae can be observed and measured as a response to stress and inflammation as demonstrated by Lepiller et al. (2007) whereby the NO levels measured from induced injury or LPS stimulated zebrafish larvae were considerably higher than that of the control. This direct method of visualizing and quantifying an inflammatory response within a living vertebrate is fast gaining popularity as a rapid and relatively simple assay and this has subsequently led to several anti-inflammatory studies of natural products (Lee, 2017; Gutierrez et al., 2018; Zou et al., 2019). Likewise, in this study, the optically transparent zebrafish larvae was utilized to observe the potential anti-inflammatory effects that NDOs have on zebrafish larvae when treated with lipopolysaccharide (LPS) from S. Enteritidis. The LPS induced NO was measured based on the increase in fluorescence intensity of the heart as studies have reported an appreciable increase in NO in cardiac muscles in response to bacterial endotoxin (Balligand and Cannon, 1997; Flesch et al., 1999).

One of the NDOs used will be oligosaccharides extracted from palm kernel cake (PKC) as these PKC oligosaccharides (OligoPKC), particularly those of the lower degree of polymerization, has been shown to exhibit prebiotic properties (Rezaei et al., 2015; Jahromi et al., 2016; Foo et al., 2020). In addition to that, Jahromi Faseleh et al. (2017) has also reported that oligosaccharides from PKC were able to improve the antioxidant capacity of the liver in rats. With NO itself being a free radical that causes harm if produced in excess, it would be interesting to observe the reported antioxidant properties of PKC in action in real-time through the use of a fluorescence microscope using zebrafish as an animal model. Two other commercial NDOs, namely mannanoligosaccharides (MOS) and fructooligosaccharides (FOS) were included as comparison in this study. Prior to the experiment, an acute toxicity test was carried out to determine an appropriate dose of NDOs to provide to the zebrafish larvae.

## Materials and Methods

### OligoPKC Preparation

The preparation of OligoPKC is based on the method reported by Foo et al. (2020) and Jahromi et al. (2016). Commercial oligosaccharides MOS (Henan Junda Biological Technology Co., Ltd., China) and FOS (Quantum Hi-Tech (China) Biological Co., Ltd., China) were used as comparison to OligoPKC in this study.

### Zebrafish Care and Maintenance

All procedures involving zebrafish embryos and larvae have been approved and are in compliance with the Institutional Animal Care and Use Committee (IACUC) guidelines on behalf of Universiti Putra Malaysia. AUP No.: UPM/IACUC/AUP-R064/2019.

### Zebrafish Embryo Acute Toxicity Test

Zebrafish acute toxicity kits (Catalog number: DAL-ZFET-AM01) complete with the necessary amount of (wild-type short fin) embryos needed to conduct the assay were purchased from Danio Assay Laboratories Sdn Bhd, Malaysia. Upon receiving the kits and the embryos, the fertilization time of the embryos was noted and the dead embryos were discarded and the medium was replaced with fresh E3 embryo medium containing 0.1% DMSO (supplied). The live embryos were then stored at 28 ± 2°C until they reached the pharyngula stage [24 h post-fertilization (hpf)]. At 24 hpf, embryo selection was done and only viable embryos without deformities were placed into 96-well plates (1 embryo per well) for the acute toxicity testing. The oligosaccharide concentration used ranged from 15.63 to 1,000 μg of dissolved oligosaccharide in 1 ml of E3 embryo medium containing 0.1% DMSO (μg/ml). Testing procedures were carried out based on the kit’s protocol and the embryos were monitored for 120 h. Observations were done using a Dino-Eye Edge Eyepiece Camera equipped with the DinoCapture 2.0 image processing software (AM7025X, United States) every 24 h for: (i) survival rate; (ii) hatching rate; and (iii) malformation.

### *In vivo* Measurement of Nitric Oxide Levels in Zebrafish Larvae

Nitric oxide levels in zebrafish larvae in response to LPS-induced stress was done according to Lepiller et al. (2007) with some modifications. Zebrafish embryos (Danio Assay Laboratories, Malaysia) were kept in a petri dish containing autoclaved E3 embryo medium with 0.003 mg/ml N-phenylthiourea (PTU) (Sigma-Aldrich, United States). Temperature was maintained at 28 ± 2°C. E3 embryo medium was changed every day and each petri dish contained approximately 100 embryos.

At 5 days post fertilization (dpf) larvae were transferred into 24-well plates containing E3 embryo medium with 0.003 mg/ml PTU and their respective treatments for 48 h. Treatments consists of either: (i) no oligosaccharides or LPS; (ii) 250 μg/ml of oligosaccharides; (iii) a mixture of oligosaccharides and LPS from SE (30 μg/ml) and; (iv) LPS from *S.* Enteritidis (30 μg/ml) alone. Oligosaccharides tested were either OligoPKC, MOS or FOS. After 48 h, the larvae were rinsed thrice with E3 medium and placed into E3 medium containing 5 μM 4-amino-5-methylamino-2’,7’-difluorofluorescein diacetate (DAF-FM DA) (Invitrogen, United States) for 2 h in the dark. After 2 h, the larvae were rinsed thrice once more and anesthetized with 0.168 mg/ml tricaine (Sigma-Aldrich, United States). The larvae were then transferred to 96-well plates (one larvae per well). To keep the larvae in place, 50 μl of 2.25% methycellulose (Danio Assay Laboratories Sdn Bhd, Malaysia) containing 0.168 mg/ml tricaine was added to each well. Observations were made using an inverted fluorescence microscope (Olympus IX73, Japan) with an excitation wavelength of 490 nm and an emission wavelength of 520 nm. Exposure times of 121.1 ms and 4× objective lens magnification was used. Image capture was done using cellSens Dimension software (Olympus, Japan) and fluorescence intensities of the heart (area as indicated in Supplementary Figure 2) was measured in grayscale using ImageJ software (NIH, US). The corrected total cell fluorescence (CTCF) of the heart was calculated using the following equation:

CTCF = Integrated Density - (Area of the heart × Mean fluorescence of background readings)

### Statistical Analysis

Prism Graph Pad software version 5.01 was used to run the statistical analysis. In order to test for normality, the Shapiro-Wilk test was done on all data sets (Supplementary Tables 4–7). As the data was not normally distributed, Kruskal-Wallis test followed by Dunn’s Multiple Comparison Test was used to compare each treatment to the untreated control in order to determine statistical significance. The graphical method was used to calculate the LC_50_ value of OligoPKC. Each experiment was replicated three times and the results shown are the mean values ± SEM where *p* < 0.05 was considered significant. For the acute toxicity assay, n = 10 fishes (10 fishes × 8 oligosaccharide concentrations × 3 biological replicates = 240 fishes per oligosaccharide tested). For the effects of NDOs on LPS induced zebrafish larvae, *n* = 6 fishes (6 fishes × 8 treatments × 3 biological replicates = 144 fishes).

## Results

### Acute Toxicity Assay

#### Survival Rate

**FIGURE 1:**
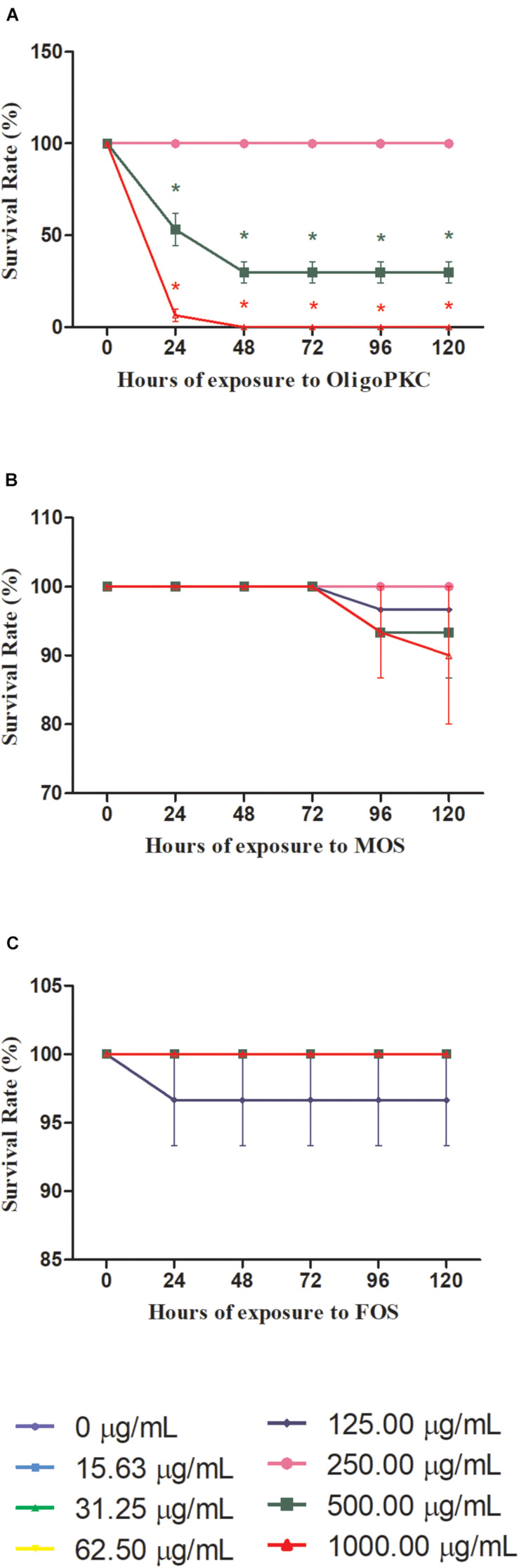
Survival rates of zebrafish embryos treated with different concentrations of **(A)** OligoPKC; **(B)** MOS; and **(C)** FOS. Each plotted value represent the mean ± SEM of three biological replicates with each biological replicate containing (*n* = 10) fishes. Statistical analysis was done using Kruskal-Wallis test followed by Dunn’s Multiple Comparison Test. Means with the symbol “*” are considered significantly different (*p* ≤ 0.05) compared to the untreated control. Data points appear overlapped at 100% survival rate.

Of the three oligosaccharides tested for an exposure period of 120 h, the OligoPKC showed the lowest survival rate amongst the zebrafish embryos with 46.67% of the deaths occurring at 500 μg/ml and 93.3% at 1,000 μg/ml just after 24 h of exposure. The death toll for 500 μg/ml further increased to 70.0% after 48 h of exposure but subsequently stabilized with no further mortality up to 120 h. At 1,000 μg/ml OligoPKC, 100% mortality of zebrafish embryos we observed within 48 h of exposure. From the graph of mortality vs. base 10 logarithm (log_10_) of OligoPKC concentration (Supplementary Figure 1), the obtained LC_50_ for the OligoPKC is 488.1 μg/ml. There was no mortality observed in zebrafish embryos treated with OligoPKC up to a concentration of 250 μg/ml. These data points overlap one another at 100% survivability in Figure 1A. Embryos treated with commercial MOS (Figure 1B) showed no mortality up to 72 h. At 96 h, deaths were observed from groups exposed to 1,000, 500, and 125 μg/ml MOS but the mortality was statistically significant (*P* < 0.05) only at the 120 h with a 10% mortality rate for 1,000 μg/ml MOS. As the survival rate for MOS remained above 50%, the LC_50_ for MOS, up to 1,000 μg/ml, could not be calculated. Only a single death in one out of the three biological replicates was observed for embryos treated with 125 μg/ml commercial FOS (Figure 1C). The death of the single embryo was not statistically significant indicating that up to 1,000 μg/ml, FOS had no toxicity effects on zebrafish embryos.

#### Hatching Rate

**FIGURE 2:**
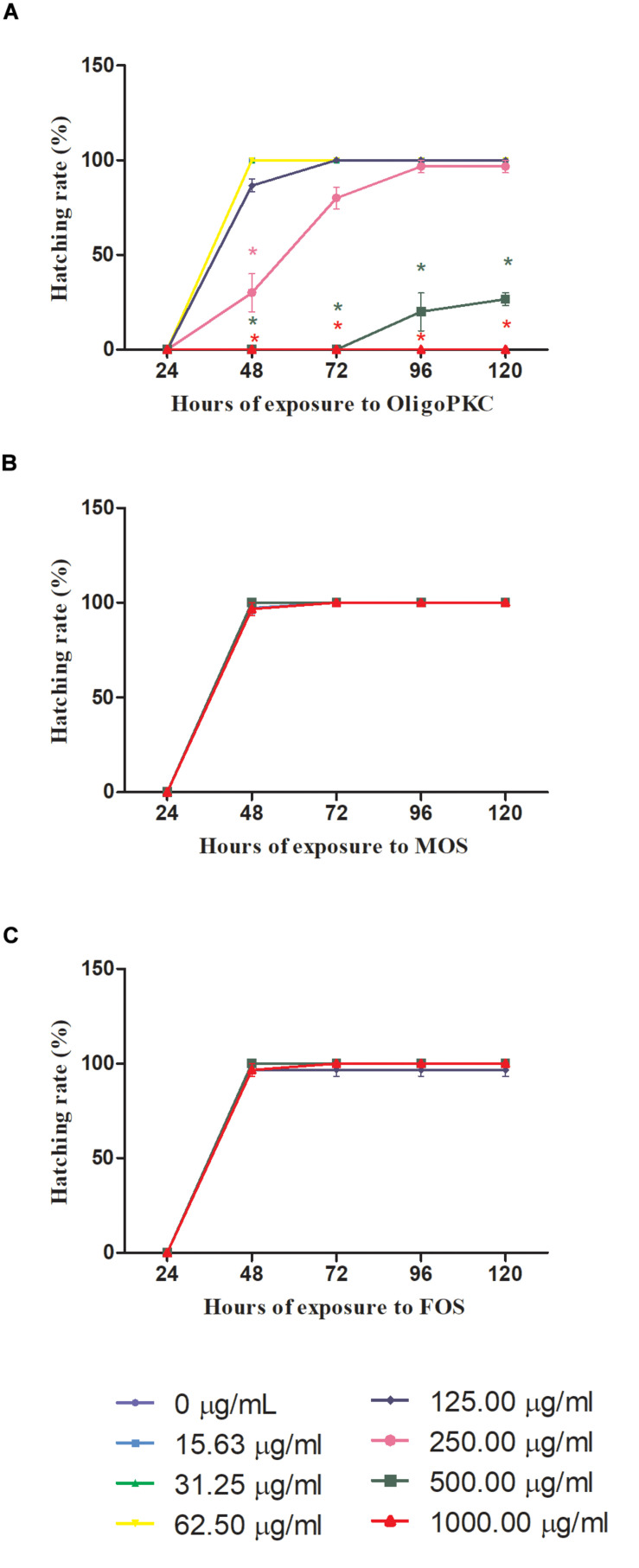
Hatching rates of zebrafish embryos treated with different concentrations of **(A)** OligoPKC; **(B)** of MOS and; **(C)** FOS. Each plotted value represent the mean ± SEM of three biological replicates with each biological replicate containing (n = 10) fishes. Statistical analysis was done using Kruskal-Wallis test followed by Dunn’s Multiple Comparison Test. Means with the symbol “*” are considered significantly different (*p* ≤ 0.05) compared to the untreated control. Data points appear overlapped at 100% hatching rate.

The results in Figure 2 showed that 48 h is needed for the control (untreated) embryo to achieve 100% hatching rate. Figure 2A shows the effect of OligoPKC on the hatching rates of zebrafish embryos. When treated with 125 μg/ml of OligoPKC, 100% hatching rate was achieved after 72 h of exposure; a delay of 24 h. No deaths were recorded for embryos treated with 250 μg/ml of OligoPKC with 96.7% of the embryos hatched by the final observation point at 120 h. A further delay and inhibition of hatching was observed for 500 μg/ml OligoPKC treatment with only 20.0 and 26.7% of the embryos hatched by the 96 and 120 h time points, respectively, despite a survival rate of 30%. No hatchings were recorded for embryos treated with 1,000 μg/ml OligoPKC as all embryos were dead within 24 h of exposure.

MOS and FOS (Figures 2B,C) treatments did not affect hatching rates of the zebrafish embryos. With MOS, all embryos were hatched by the 72 h time point and for FOS treated embryos; a 100% hatching rate was achieved by the 48 h observation point with one death recorded for 125 μg/ml.

#### Malformation Effects

**FIGURE 3:**
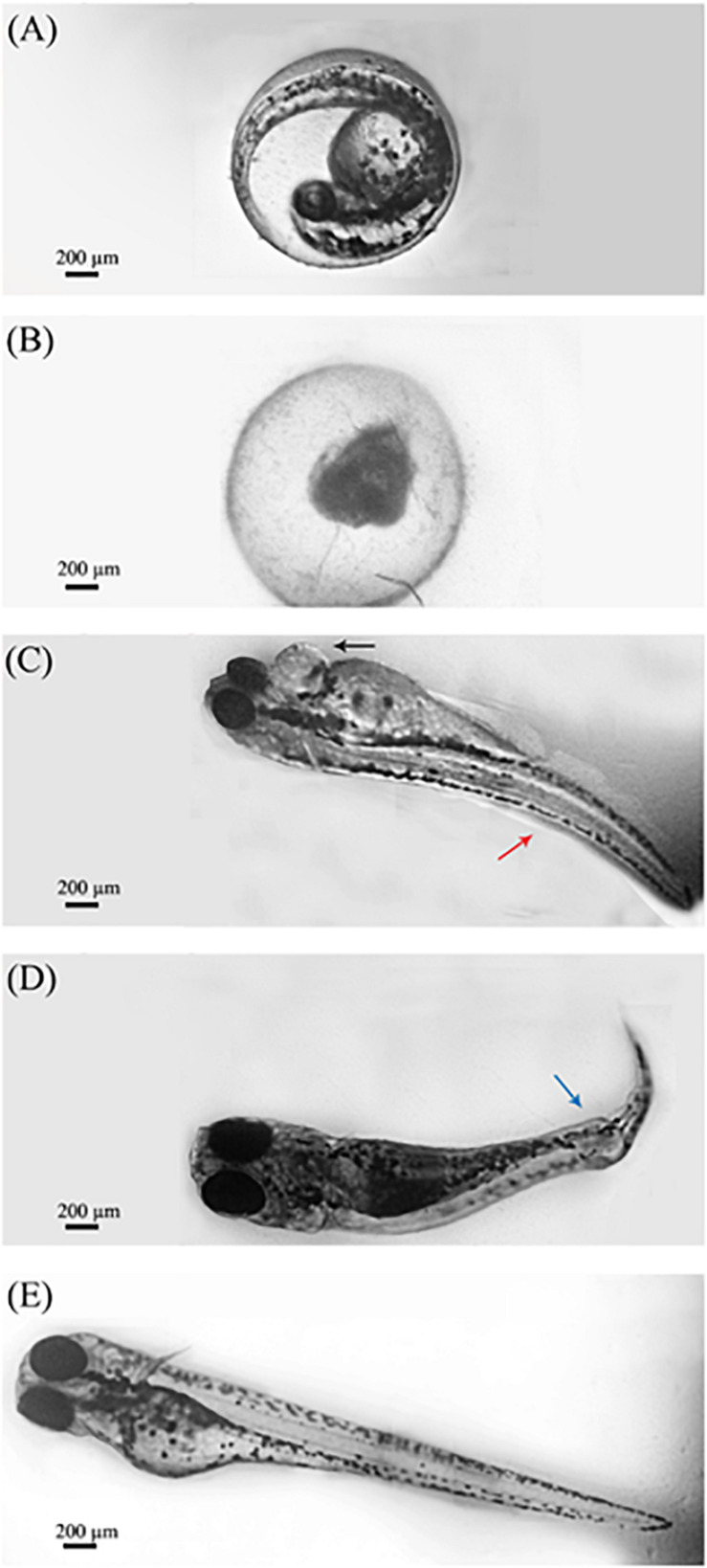
Morphological characteristics of zebrafish larvae after exposure to OligoPKC. Image capture was done through a 4× objective lens. **(A)** Zebrafish embryo observed at the pharyngula stage (24 hpf) without any OligoPKC treatment and 0 h of exposure. **(B)** Lethal toxicity of OlicoPKC at 1,000 μg/ml. Larvae at 6 dpf appear unhatched and coagulated after 120 h of exposure; **(C)** zebrafish larvae at 6 dpf showing pericardial edema (black arrow) and body curvature (red arrow) at 500 μg/ml after 120 h of exposure; **(D)** zebrafish larvae at 6 dpf showing kinked tail (blue arrow) at 500 μg/ml after 120 h of exposure and; **(E)** control. Larvae at 6 dpf appears normal and is free of developmental defects. Image processing using Adobe Photoshop CS3 have been applied in order to group several snapshots of the same larvae into one cohesive image and to reduce the background noise of the images in question. The changes made to the images does not alter the intended information shown in the figure. Scale bar = 200 μm.

Figure 3 shows the representative teratogenic effects of OligoPKC on 6 dpf zebrafish larvae after 120 h of exposure. Treatment of zebrafish embryos with OligoPKC at the highest concentration (1,000 μg/ml) resulted in lethal toxicity within 48 h of exposure. As a result of that, further development was halted and the expired embryos remained within their chorions as shown in Figure 3A. While not as lethal, the treatment of zebrafish embryos with 500 μg/ml OligoPKC led to developmental malformations after 96 h of exposure (Figures 3B,C). At the 96 h observation point, only 30.0% of the zebrafish embryos remained viable and out of those that were viable, 44.4% showed developmental abnormalities such as pericardial edema, body curvature and kinked tail. At 31.25 and 250 μg/ml OligoPKC, a single larvae out of the three biological replicates developed yolk sac edema after 24 and 120 h of exposure, respectively. The occurrence of a single larvae with yolk sac edema was not considered statistically significant. OligoPKC at concentrations of 125.0, 62.5, and 15.63 μg/ml showed no teratogenic effects. Likewise, no teratogenic effects were observed in zebrafish embryos treated with MOS or FOS up to 1,000 μg/ml.

### Measurement of *in vivo* Nitric Oxide Levels

As OligoPKC at 250 μg/ml showed no lethal or malformation effects, this was the treatment level chosen for all NDOs for the following study. The *in vivo* quantification of cardiac NO levels in zebrafish larvae is depicted in Supplementary Figures 2, 3. A higher fluorescence signal, is indicative of higher NO production and this is apparent when LPS at 30 μg/ml induces a significantly higher fluorescence signal in zebrafish larvae when compared to the untreated control (Supplementary Figure 3).

**FIGURE 4:**
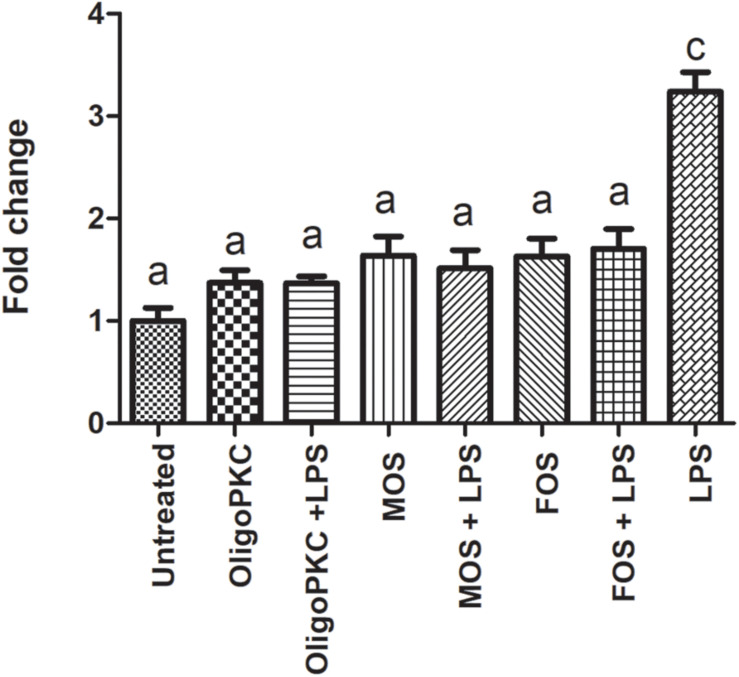
Fluorescence intensity of zebrafish larvae at 7 dpf treated with OligoPKC, MOS, FOS, a combination of different oligosaccharides and LPS. The values represent the mean ± SEM of three biological replicates with each biological replicate containing (*n* = 6) fishes. Statistical analysis was done using Kruskal-Wallis test followed by Dunn’s Multiple Comparison Test. Means with the same letter are not statistically significant (*p* > 0.05) from each other.

The results presented in Figure 4 and Supplementary Table 2 shows the effects of OligoPKC on the production of NO with and without the presence of LPS. The addition of 250 μg/ml of OligoPKC did not significantly increase NO production in zebrafish larvae. When given in combination with LPS, the mixture of OligoPKC and LPS had a significantly lower NO level compared to zebrafish larvae treated with only LPS. This significant reduction in NO levels, when NDOs are given in combination with LPS were also observed in larvae treated commercial MOS and, to a lesser degree, FOS at 250 μg/ml.

## Discussion

Of the three oligosaccharides tested, FOS was found to be non-toxic up to 1,000 μg/ml for zebrafish embryos up to 120 h of exposure. These findings were within expectations as FOS, which is an established prebiotic should not cause any adverse reaction to the consumer (Roberfroid et al., 2010). The mortality was observed in zebrafish embryos after 120 h of exposure at 1,000 μg/ml could likely stem from the immunogenic properties of yeast mannan (which is a polymer of the sugar mannose) (Ballou, 1970). Immunogenicity, which is the ability of a substance to trigger an immune response, may be useful for rapidly resolving an infection. However, prolonged exposure at high concentrations to such a substance may be counterproductive in the long run and may even lead to toxicity (Tovey and Lallemand, 2011); as may be the case for the commercial MOS.

For OligoPKC, the fraction at 500 μg/ml and above exhibited both acute toxicity and teratogenicity effects on the zebrafish embryos involved. HPLC analysis from our laboratory and literature reported that mannose makes up a large portion of the oligosaccharides extracted from PKC and it is the low degree of polymerization (DP) molecule portion, such as those found in the OligoPKC used in this study that exerts its bioactive properties (Ibuki et al., 2011; Jahromi et al., 2016; Foo et al., 2020). However the more potent the biological effect, the higher the toxicity. Such an outcome has been reported in a similar study done by Zou et al. (2019) whereby agaro-oligosaccharide fractions with the highest anti-inflammatory activity also exhibited the highest cytotoxicity. The difference in toxicity and teratogenicity observed in OligoPKC when compared to MOS could be due to the oligosaccharide composition as well as structure. In terms of oligosaccharide composition, the saccharides present in the OligoPKC used in this study were up to 6 DP while the DP of the purchased MOS was not specified by the manufacturer (Henanjunda, 2017; Foo et al., 2020). Molecular weight determination of MOS using Liquid Chromatography Quadrupole Time-of-Flight Mass Spectrometry (LC-QTOF/MS) indicates that the saccharides present in MOS may be larger than 10 DP or that these saccharides may exist as oligosaccharide derivatives (Lesage and Bussey, 2006; Foo et al., 2020). In terms of chemical structure, the mannan oligosaccharide extracted from PKC is of the β-configuration while the chemical structure of the MOS extracted from the yeast cell wall is of the α-configuration (Jones and Ballou, 1969; Jahromi et al., 2016). Even amongst mannan extracted from different strains of yeast, the immunogenic effects elicited may differ significantly depending on the pattern of the side chains involved (Ballou, 1970).

Due to the embryotoxicity effects of OligoPKC, it was decided that 250 μg/ml would be used for subsequent experiments as there were no mortality or statistically significant teratogenic effects observed in embryos at that concentration. Lipopolysaccharide at a concentration of 30 μg/ml was used to trigger the generation of NO in zebrafish embryos. The fluorescence probe, DAF-FM-diacetate, used in this study to detect NO *in vivo* works by permeating through living cells and once inside the cells, the DAF-FM-diacetate reacts with NO to form a fluorescent triazole derivative with the aid of intracellular esterases (Lepiller et al., 2007). This fluorescent signal produced appears to be proportional to the amount of NO present as evidenced in Supplementary Figure 3 and Supplementary Table 1. One of the symptoms of LPS induced septic shock is the large amount of NO generated by cardiac muscle cells (Titheradge, 1999). These excessive levels of NO could lead to not only hypotension but multiple organ dysfunction as well (Titheradge, 1999).

Non-digestible oligosaccharides have been reported to reduce oxidative stress and inflammation through the direct scavenging of intracellular radicals as well as through the modulation of inflammatory responses in the host (Ngo et al., 2009; Kothari et al., 2014; Wu et al., 2017). In a study conducted by Jahromi Faseleh et al. (2017), the use of oligosaccharides from PKC with a DP range of two to eight and a concentration of 10 mg/ml was capable of downregulating the expressions of *IL-1*β, *IL-2*, and monocyte chemoattractant protein-1 (*MCR-1*); upregulating the expressions of *IL-10, IFN-*γ, and *TNF-*α; and improving the antioxidant capacity of the liver in rats. At a glance, the present findings (Figure 4) which shows a reduction in cardiac NO levels are in agreement with the antioxidant effects mentioned in the above studies. While it is good that none of the oligosaccharides tested caused a significantly higher increase in NO levels when compared to the untreated control, of notable interest is that when a mixture of oligosaccharide and LPS is administered, the NO levels of all three groups were lower than that LPS alone indicating that NDOs were capable of reducing the NO levels in the presence of LPS. As MOS and FOS were non-toxic up to 1,000 μg/ml, these NDOs were also tested for their NO reducing effects at 1,000 μg/ml as shown in Supplementary Figure 4 and Supplementary Table 3. At 1,000 μg/ml, the NO reducing effects of MOS showed no significant change from its effect at 250 μg/ml while FOS at 1,000 μg/ml was comparable to OligoPKC and MOS at 250 μg/ml.

Possible explanations for the observed decreased NO levels in groups treated with a mixture of oligosaccharide and LPS could be linked to the oligosaccharides’ ability to downregulate Toll-like receptor 4 (TLR4) signaling which in turn reduces host sensitivity to LPS and subsequently causing a reduction in NO levels (Salazar et al., 2016; Wu et al., 2017). However, future studies involving the elucidation of NDOs mechanism of action would be needed to confirm this hypothesis.

## Conclusion

In conclusion, the use of DAF-FM-diacetate as a fluorescence probe to detect changes in NO levels in zebrafish larvae shows potential as a non-invasive, relatively straightforward and rapid end-point assay and may be included as part of a multicomponent analysis to study inflammation. The use of OligoPKC to reduce NO in LPS stimulated zebrafish larvae was comparable to that of the commercial oligosaccharides MOS and better than FOS at 250 μg/ml. However its upper limit is restricted due to the toxicity exhibited at 500 μg/ml and above but in this case, 250 μg/ml appears sufficient in significantly reducing LPS induced NO without the need for a higher NDOs concentration.

## Data Availability Statement

All datasets generated for this study are included in the article/Supplementary Material.

## Ethics Statement

The animal study was reviewed and approved by the Institutional Animal Care and Use Committee, Universiti Putra Malaysia. AUP No.: UPM/IACUC/AUP-R064/2019.

## Author Contributions

ZI and JL were responsible for funding and resource acquisition. JL, ZI, SA, KL, KY, and RF were involved with supervision and experimental design. RF carried out the experiments, data analysis, and manuscript writing. All authors have read and approved of the manuscript for submission.

## Conflict of Interest

The authors declare that the research was conducted in the absence of any commercial or financial relationships that could be construed as a potential conflict of interest.

## Supplementary Material

Supplementary Figure 1 Graph of survival rate (%) of zebrafish larvae after 120 h of exposure vs. base 10 logarithm (log_10_) of OligoPKC. Each plot represents the mean values ± SEM of three biological replicates with *n* = 10 larvae per OligoPKC concentration per biological replicate.

Supplementary Figure 2 Effect of different levels of LPS on the fluorescent intensities (indicative of NO levels) in the heart region (encircled in white) of zebrafish larvae at 5 dpf with a 4× objective lens magnification. **(A)** untreated control; **(B)** 20 μg/ml LPS; **(C)** 25 μg/ml LPS and; **(D)** 30 μg/ml LPS. The fold change of fluorescent intensities is presented in Supplementary Table 1. Image processing using the “Smart Sharpen” filter on Adobe Photoshop CS3 has been applied uniformly on all four images in order to improve image clarity. The changes made to the images do not alter the information conveyed in the figure. Scale bar = 200 μm.

Supplementary Figure 3 Preliminary study involving the measurement of fluorescence intensity of zebrafish larvae at 5 dpf which corresponds to different concentrations of LPS from Supplementary Figure 2 and Supplementary Table 1. Statistical analysis was done using Kruskal-Wallis test followed by Dunn’s Multiple Comparison Test. The values represent the mean ± SEM of three larvae. Means with the same letter are not statistically significant (*p* > 0.05) from each other.

Supplementary Figure 4 Fluorescence intensity of zebrafish larvae at 7 dpf treated with MOS, FOS, a combination of different oligosaccharides and LPS. The concentration of MOS and FOS used is 1,000 μg/ml. The values represent the mean ± SEM of three biological replicates with each biological replicate containing (*n* = 6) fishes. Statistical analysis was done using Kruskal-Wallis test followed by Dunn’s Multiple Comparison Test. Means with the same letter are not statistically significant (*p* > 0.05) from each other. The fold change values of the Untreated and LPS are the same as that shown in Supplementary Table 2.

Supplementary Table 1 Fold changes of fluorescent intensities in zebrafish larvae in response to different concentrations of LPS at 5 dpf. Each value represents the fold change in fluorescent intensity for a single larvae with *n* = 3 larvae per treatment. Statistical analysis was done Kruskal-Wallis test followed by Dunn’s Multiple Comparison Test and is presented in Supplementary Figure 3.

Supplementary Table 2 Fold changes of fluorescent intensities in zebrafish larvae in response to different treatment groups. Each value represents the fold change in fluorescent intensity for a single larvae at 7 dpf. Each treatment contains three biological replicates and each biological replicate in turn consist of *n* = 6 larvae. Statistical analysis was done using Kruskal-Wallis test followed by Dunn’s Multiple Comparison Test and is presented in Figure 4.

Supplementary Table 3 Fold changes of fluorescent intensities in zebrafish larvae in response to different treatment groups of MOS and FOS at 7 dpf. The concentration of MOS and FOS used is 1,000 μg/ml. Each value represents the fold change in fluorescent intensity for a single larvae. Each treatment contains three biological replicates and each biological replicate in turn consist of *n* = 6 larvae. Statistical analysis was done using Kruskal-Wallis test followed by Dunn’s Multiple Comparison Test and is presented in Supplementary Figure 4. The fold change vales of the Untreated and LPS are the same as that shown in Supplementary Table 2.

Supplementary Table 4 Shapiro-Wilk test for normality for survival rates of zebrafish larvae after 24 h of exposure to OligoPKC. Total number of zebrafishes, *n* = 10 (10 fishes × 3 biological replicate = 30).

Supplementary Table 5 Shapiro-Wilk test for normality for hatching rates of zebrafish larvae after 48 h of exposure to OligoPKC. Total number of zebrafishes, *n* = 10 (10 fishes × 3 biological replicate = 30).

Supplementary Table 6 Shapiro-Wilk test for normality for fold changes of fluorescence intensity of zebrafish larvae at 7 dpf treated with OligoPKC, MOS, FOS, a combination of different oligosaccharides and LPS. Total number of zebrafishes, *n* = 6 fishes (6 fishes × 8 treatments × 3 biological replicates = 144 fishes).

Supplementary Table 7 Shapiro-Wilk test for normality for fold changes of fluorescent intensities in zebrafish larvae in response to different treatment groups of MOS and FOS at 7 dpf. The concentration of MOS and FOS used is 1,000 μg/ml. The fold change values of the Untreated and LPS are the same as that shown in Supplementary Table 2. Total number of zebrafishes, *n* = 6 fishes (6 fishes × 6 treatments × 3 biological replicates = 108 fishes).
