# Supplementary material for: Palm Kernel Cake Oligosaccharides Acute Toxicity and Effects on Nitric Oxide Levels Using a Zebrafish Larvae Model
Source: Front Physiol. 2020 Sep 24;11:555122. doi: 10.3389/fphys.2020.555122 (PMC7541901; doi:10.3389/fphys.2020.555122)
Supplement: Supplementary Figure 1 — Graph of survival rate (%) of zebrafish larvae after 120 h of exposure vs. base 10 logarithm (log10) of OligoPKC. Each plot represents the mean values ± SEM of three biological replicates with n = 10 larvae per OligoPKC concentration per biological replicate. [file Data_Sheet_1.pdf]

Supplementary Material

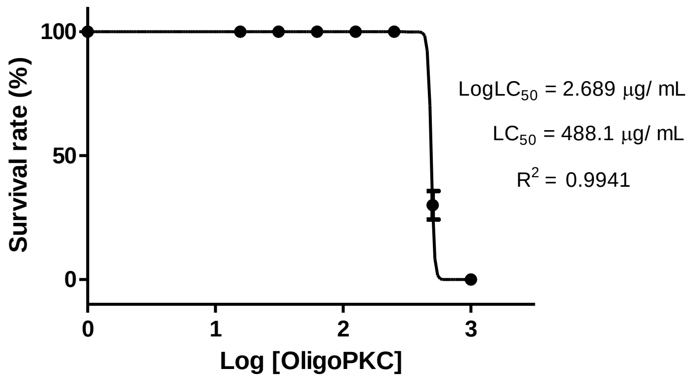

Supplementary Figure 1. Graph of survival rate (%) of zebrafish larvae after 120 hours of exposure versus base 10 logarithm (log_{10}) of OligoPKC. Each plot represents the mean values ± SEM of three biological replicates with n=10 larvae per OligoPKC concentration per biological replicate.

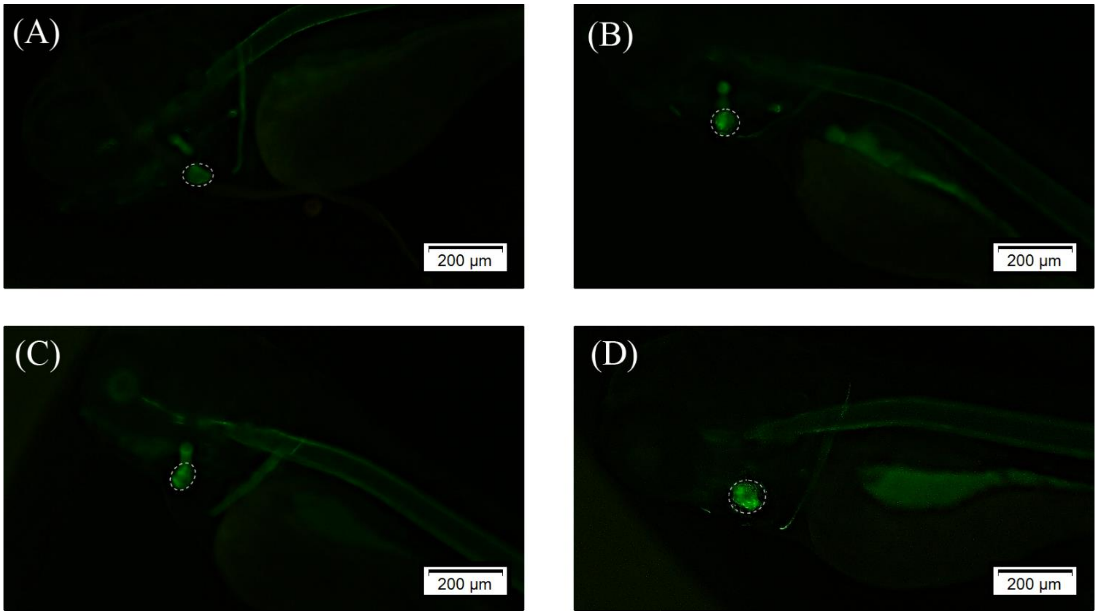

Supplementary Figure 2. Effect of different levels of LPS on the fluorescent intensities (indicative of NO levels) in the heart region (encircled in white) of zebrafish larvae at 5 dpf with a 4x objective lens magnification(a) untreated control; (b) 20 µg/ml LPS; (c) 25 µg/ml LPS and; (d) 30 µg/ml LPS. The fold change of fluorescent intensities is presented in Supplementary Table 1. Image processing using the 'Smart Sharpen' filter on Adobe Photoshop CS3 has been applied uniformly on all four images in order to improve image clarity. The changes made to the images do not alter the information conveyed in the figure. Scale bar = 200 µm.

Supplementary Table 1. Fold changes of fluorescent intensities in zebrafish larvae in response to different concentrations of LPS at 5 dpf. Each value represents the fold change in fluorescent intensity for a single larvae with n=3 larvae per treatment Statistical analysis was done using Kruskal-Wallis test followed by Dunn's Multiple Comparison Test and is presented in Supplementary Figure 3.

Fold change of fluorescent intensities			
Control	20 μg/ml	25 μg/ml	30 μg/ml
0.835707	1.826024	2.312948	2.83227
1.2888	1.77892	2.265107	3.478989
0.875492	1.889671	1.843199	2.947752

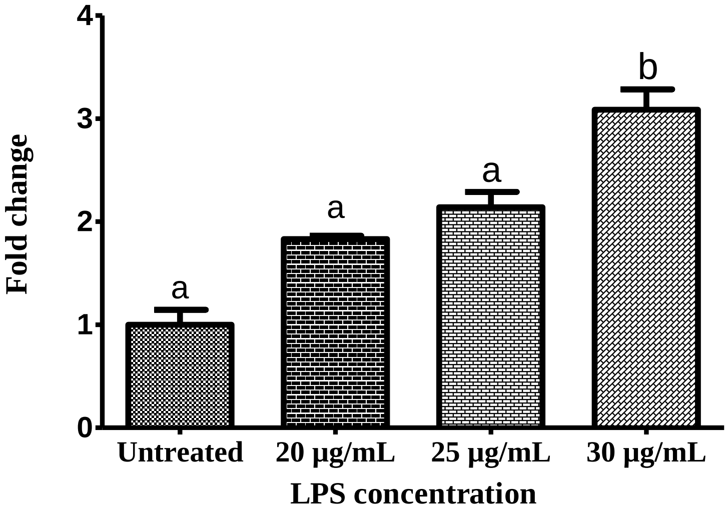

Supplementary Figure 3. Preliminary study involving the measurement of fluorescence intensity of zebrafish larvae at 5 dpf which corresponds to different concentrations of LPS from Supplementary Figure 2 and Supplementary Table 1. Statistical analysis was done using Kruskal-Wallis test followed by Dunn's Multiple Comparison Test. The values represent the mean ± SEM of three larvae. Means with the same letter are not statistically significant ($p > 0.05$) from each other.

Supplementary Table 2. Fold changes of fluorescent intensities in zebrafish larvae in response to different treatment groups. Each value represents the fold change in fluorescent intensity for a single larvae at 7 dpf. Each treatment contains three biological replicates and each biological replicate in turn consist of n=6 larvae. Statistical analysis was done using Kruskal-Wallis test followed by Dunn's Multiple Comparison Test and is presented in Figure 4.

	Fold change of fluorescent intensities							
	Untreated	OligoPKC	OligoPKC +LPS	MOS	MOS + LPS	FOS	FOS + LPS	LPS
Biological replicate 1	0.72	1.622	1.537	1.411	1.775	1.855	2.709	3.326
	0.733	1.326	0.947	1.358	1.269	1.412	1.619	2.23
	0.677	1.272	1.078	1.544	1.285	1.231	0.903	2.352
	0.462	1.444	1.394	3.447	3.443	2.092	1.333	2.838
	0.377	0.966	1.157	2.816	2.575	1.932	0.724	2.943
	0.53	0.986	1.204	2.779	2.964	3.387	0.866	2.702
Biological replicate 2	1.738	1.423	1.726	1.039	0.921	0.746	1.978	3.166
	2.018	1.352	1.772	1.472	1.282	1.164	2.272	3.463
	1.108	2.427	1.15	1.186	1.222	2.465	1.865	4.033
	1.866	0.609	1.169	0.947	1.413	1.79	1.553	4.147
	1.4	2.577	1.543	0.996	1.161	2.124	0.952	4.213
	1.822	2.073	1.44	1.109	0.91	1.045	0.886	4.787
Biological replicate 3	0.418	0.759	1.21	2.081	1.954	1.792	1.674	2.34
	0.968	1.184	1.351	2.046	0.892	2.632	2.85	2.138
	0.566	1.297	1.752	2.092	0.884	1.308	3.711	3.874
	0.857	1.426	1.344	0.204	0.822	0.941	2.356	3.393
	1.152	0.9	0.972	1.679	1.329	0.835	1.262	3.996
	0.596	1.023	1.899	1.301	1.147	0.638	1.206	2.346

Supplementary Table 3. Fold changes of fluorescent intensities in zebrafish larvae in response to different treatment groups of MOS and FOS at 7 dpf. The concentration of MOS and FOS used is 1000 µg/ml. Each value represents the fold change in fluorescent intensity for a single larvae. Each treatment contains three biological replicates and each biological replicate in turn consist of n=6 larvae. Statistical analysis was done using Kruskal-Wallis test followed by Dunn's Multiple Comparison Test and is presented in Supplementary Figure 4. The fold change vales of the Untreated and LPS are the same as that shown in Supplementary Table 2.

	Fold change of fluorescent intensities					
	Untreated	MOS	MOS + LPS	FOS	FOS + LPS	LPS
Biological replicate 1	0.72	1.447	1.489	0.937	1.239	3.326
	0.733	1.403	1.803	1.157	1.135	2.23
	0.677	1.337	1.834	1.182	1.063	2.352
	0.462	1.368	1.925	0.886	0.866	2.838
	0.377	1.222	1.477	0.922	1.023	2.943
	0.53	1.392	1.315	1.104	1.132	2.702
Biological replicate 2	1.738	1.436	1.15	1.336	1.071	3.166
	2.018	1.544	0.885	1.237	0.93	3.463
	1.108	1.782	1.256	1.027	1.631	4.033
	1.866	1.455	0.957	1.319	1.602	4.147
	1.4	1.552	0.924	1.195	1.483	4.213
	1.822	1.63	0.93	1.026	1.509	4.787
Biological replicate 3	0.418	1.16	1.423	1.056	1.124	2.34
	0.968	1.386	1.199	1.001	1.902	2.138
	0.566	1.067	1.075	1.621	1.701	3.874
	0.857	1.241	1.141	1.882	1.054	3.393
	1.152	1.638	0.935	0.808	2.401	3.996
	0.596	1.448	1.113	1.685	2.336	2.346

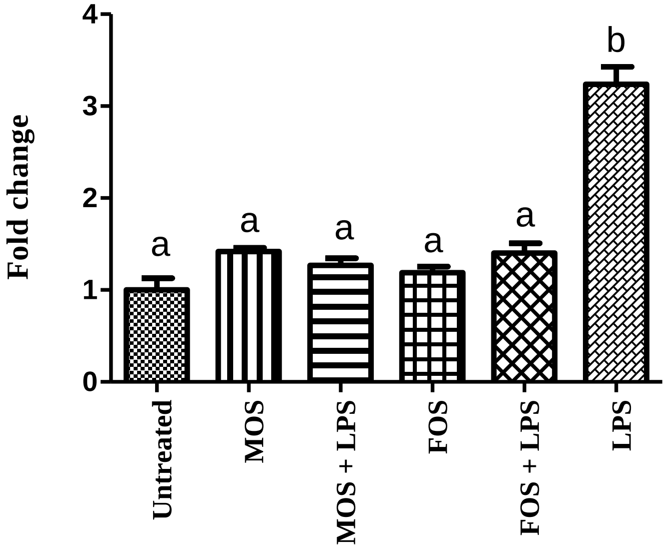

Supplementary Figure 4. Fluorescence intensity of zebrafish larvae at 7dpf treated with MOS, FOS, a combination of different oligosaccharides and LPS. The concentration of MOS and FOS used is 1000 µg/ml. The values represent the mean ± SEM of three biological replicates with each biological replicate containing (n=6) fishes. Statistical analysis was done using Kruskal-Wallis test followed by Dunn's Multiple Comparison Test. Means with the same letter are not statistically significant ($p > 0.05$) from each other. The fold change values of the Untreated and LPS are the same as that shown in Supplementary Table 2.

Supplementary Table 4. Shapiro-Wilk test for normality for survival rates of zebrafish larvae after 24 hours of exposure to OligoPKC. Total number of zebrafishes, n=10 (10 fishes x 3 biological replicate =30 fishes)

	Concentration (µg/ mL)							
	0	15.63	31.25	62.5	125	250	500	1000
W	-	-	-	-	-	-	0.6366	0.2754
P Value	-	-	-	-	-	-	< 0.0001	< 0.0001
Passed Normality test (alpha = 0.05)?	-	-	-	-	-	-	No	No
P value summary	-	-	-	-	-	-	***	***

Supplementary Table 5. Shapiro-Wilk test for normality for hatching rates of zebrafish larvae after 48 hours of exposure to OligoPKC. Total number of zebrafishes, n=10 (10 fishes x 3 biological replicate =30)

	Concentration (µg/ mL)							
	0	15.63	31.25	62.5	125	250	500	1000
W	-	-	-	-	0.404	0.577	-	-
P Value	-	-	-	-	< 0.0001	< 0.0001	-	-
Passed Normality test (alpha = 0.05)?	-	-	-	-	No	No	-	-
P value summary	-	-	-	-	***	***	-	-

Supplementary Table 6. Shapiro-Wilk test for normality for fold changes of fluorescence intensity of zebrafish larvae at 7 dpf treated with OligoPKC, MOS, FOS, a combination of different oligosaccharides and LPS. Total number of zebrafishes, n=6 fishes (6 fishes x 8 treatments x 3 biological replicates = 144 fishes).

	Treatment							
	Untreated	OligoPKC	OligoPKC + LPS	MOS	MOS + LPS	FOS	FOS + LPS	LPS
W	0.8822	0.9031	0.9500	0.9418	0.7947	0.9497	0.9218	0.9412
P Value	0.0284	0.0650	0.4246	0.3107	0.0013	0.4205	0.1394	0.3033
Passed Normality test (alpha = 0.05)?	No	Yes	Yes	Yes	No	Yes	Yes	Yes
P value summary	*	ns	ns	ns	**	ns	ns	ns

Supplementary Table 7. Shapiro-Wilk test for normality for fold changes of fluorescent intensities in zebrafish larvae in response to different treatment groups of MOS and FOS at 7 dpf. The concentration of MOS and FOS used is 1000 µg/mL. The fold change values of the Untreated and LPS are the same as that shown in Supplementary Table 2. Total number of zebrafishes, n=6 fishes (6 fishes x 6 treatments x 3 biological replicates = 108 fishes)

	Treatment					
	Untreated	MOS	MOS + LPS	FOS	FOS + LPS	LPS
W	0.882	0.981	0.900	0.904	0.876	0.941
P Value	0.0284	0.9636	0.0570	0.0673	0.0226	0.3035
Passed Normality test (alpha = 0.05)?	No	Yes	Yes	Yes	No	Yes
P value summary	*	ns	ns	ns	*	ns
